# New Mutations in the 5′ Region of the *Notch* Gene Affect *Drosophila melanogaster* Oogenesis

**DOI:** 10.3390/jdb10030032

**Published:** 2022-08-09

**Authors:** Elena I. Volkova, Natalya V. Dorogova, Oleg V. Andreyenkov, Saveliy A. Tikhomirov, Sergey A. Demakov

**Affiliations:** 1 Institute of Molecular and Cellular Biology, Siberian Branch of Russian Academy of Sciences (SB RAS), 630090 Novosibirsk, Russia; 2 Institute of Cytology and Genetics, Siberian Branch of Russian Academy of Sciences (SB RAS), 630090 Novosibirsk, Russia

**Keywords:** *Drosophila*, oogenesis, *Notch* expression, egg chamber, oocyte growth

## Abstract

The Notch pathway is an important and evolutionarily conserved signaling system involved in the development of multicellular organisms. Notch signaling plays an important role in the regulation of proliferation and differentiation of many cell types. In this study, we report new aspects of *Notch* gene participation in oogenesis using our previously generated mutations. The mutations consist of an insertion of an auxiliary element of a transgene construct into the first intron of the gene and a series of directed deletions within the 5′ regulatory region of *Notch*. We showed that some of these mutations affect *Drosophila* oogenesis. This insertion, either alone or in combination with the deletion of an insulator sequence, led to lower expression of *Notch* in the ovaries. As a result, the formation of egg chambers was disturbed in middle oogenesis. These abnormalities have not been described previously and imply one more function of *Notch* in oogenesis. It can be assumed that *Notch* is associated with not only follicular epithelium morphogenesis but also cellular mechanisms of oocyte growth.

## 1. Introduction

The *Notch* gene encodes a transmembrane protein (receptor) involved in the highly conserved Notch signaling pathway [1,2,3]. In *Drosophila melanogaster*, *Notch* participates in the morphogenesis of both central and peripheral nervous systems and in the development of the eyes, wings, and segmented appendages, such as legs, antennae, and muscles [4,5]. In the adult female, *Notch* is required for multiple steps in oogenesis, including the formation of the germline stem cell niche, establishment of developmental axes of the oocyte, dorsal appendage patterning [6,7,8,9], specification and differentiation of follicle cells, their switching from the mitotic cycle to the endocycle [10,11], and migration of border cells [12,13]. Many of these functions have been genetically explored by means of mutations in the *Notch* gene—as well as genes *Delta* and *Serrate* encoding Notch protein ligands—and by ectopic expression of Delta or constitutively activated mutants of receptor *Notch* [14].

In this paper, we report new effects of *Notch* on oogenesis using our original (previously generated) mutations, which were described in our earlier work [15]. The mutations have been generated using a combination of modern techniques for directed genome editing and yielded mutant fly lines containing an insertion of auxiliary elements of a transgenic construct in the first intron of the *Notch* gene and a series of directed deletions affecting possible functional sites of the 5′ regulatory region. Some of these mutations cause various defects in eye morphology, and the strongest effects are observed when the insertion is combined with deletions that remove the sequence of a putative insulator [15].

We demonstrated here that some of these mutations influence *Drosophila* oogenesis. The insertion of auxiliary elements of the transgenic construct into the first intron of *Notch,* either alone or in combination with the deletion of the insulator sequence, led to a decrease in the expression of this gene in the ovaries. As a consequence, the formation of egg chambers (ECs) was disturbed in middle oogenesis. These abnormalities alter cellular processes that ensure the growth of oocytes and assembly of the specialized epithelium around them. These *Notch* mutant phenotypes have not been described in the literature and are suggestive of one more function of *Notch* in oogenesis.

## 2. Materials and Methods

### 2.1. Drosophila Strains

Fly lines resc[w+], d3[w+], and d3[w−] with directed mutation at the *Notch* locus were described earlier [15]; lines Oregon-R and *N^fa-swb^* were obtained from the Bloomington stock center. All flies were kept at 25 °C.

### 2.2. Immunofluorescent Staining

All staining procedures were performed according to a previously described protocol [16]. Tetramethylrhodamine (TRITC)-labeled phalloidin (Sigma-Aldrich, Saint Louis, MO, USA, cat. # P1951) was used at 1:100 dilution to visualize F-actin as described elsewhere [17]. Primary antibodies were acquired from the Developmental Studies Hybridoma Bank (DSHB): mouse anti-FAS3 (1:10 dilution, clone 7G10); mouse anti-Notch intracellular domain (1:50, C17.9C6); mouse anti-Notch extracellular domain (1:50, C458.2H); mouse anti-Orb antibody (orb4H8, DSHB), 1:30; mouse anti-LaminDmO (ADL67.10, DSHB), 1:30; rabbit anti-p-Histone H3 (Ser 28) antibody (sc-8556, Santa Cruz Biotechnology, Dallas, TX, USA) 1:100; guinea pig anti-Asterless (gift of Tomer Avidor-Reiss, Professor at University of Toledo, Toledo, OH, USA), 1:500. The secondary antibody was a goat anti-mouse IgG antibody conjugated to Alexa Fluor 488 (1:500; Thermo Fisher Scientific, Waltham, MA, USA, cat. # A28175). DAPI was employed (at 1 ng/mL) to stain nuclei. Samples were viewed under an Axioscope 2 plus microscope equipped with ApoTome (Carl Zeiss, Jena, Germany). Optical sections were combined in the LSM Image Browser version 3.5 software (Carl Zeiss) and edited in Adobe Illustrator and Photoshop CS4.

### 2.3. Ovarian Morphology Analysis and Egg Counting

To this end, newly eclosed mutant and control females were placed without males into separate vials with the standard medium. The females were incubated at 25 °C for 3 and 5 days (3- and 5-day-old females, respectively). For each mutation and for the control, ovaries from 20 females were dissected in 1× PBS, then fixed with 4% paraformaldehyde in PBS, and stained with DAPI as described previously [16]. For comparative analyses, statistical significance was calculated by Student’s *t* test.

### 2.4. Reverse-Transcription Quantitative PCR (RT-qPCR)

Ovaries (20–25 pairs) from females of the lines homozygous for one of the studied deletions and from females of wild-type line Oregon-R (control) were dissected in 1× PBS and immediately transferred to TRIzol (Invitrogen) on ice. Next, the ovaries were frozen in liquid nitrogen and stored at −70 °C. RNA was isolated by means of TRIzol (Invitrogen, Waltham, MA, USA). RNA samples were treated with DNase I according to the protocol “Removal of Genomic DNA from RNA Preparations” (ThermoFisher, Waltham, MA, USA) and immediately subjected to a reverse-transcription reaction with RevertAid reverse transcriptase (ThermoFisher). qPCR was carried out using the PCR-mix Kit (Sintol, Moscow, Russia). Primers for the qPCR were as follows: Notch-F, 5′-GATCTCCGGACTCATTTCAC-3′; Notch-R, 5′-AGAAGGCCTGCTGATTGTGC-3′; Zfrp8-F, 5′-CGAGTACAACTTTCATCGGTC-3′; and Zfrp8-R, 5′-GGCAACTGTGACCTGAGGA-3′. The results of qPCR were processed in Bio-Rad CFX Manager software.

### 2.5. Western Blot

Ovaries of adult females of each genotype were collected in 1× PBS, and then SDS-PAGE (five pairs of ovaries per lane) and Western blotting were performed as described earlier [18] with some modifications. An equal volume of 2× sample buffer with β-mercaptoethanol was added to the samples and mixed well by pipetting; the mixture was heated for 5 min and loaded onto an SDS-containing 5–15% gradient polyacrylamide gel. Proteins were separated at 250 mA for 2 h and next transferred onto a Hybond ECL membrane (Amersham Pharmacia Biotech, Amersham, UK, cat. # RPN2020D). For Western blotting, the membranes were incubated overnight at 4 °C with primary antibodies (mouse anti-NICD, DSHB, cat. # C17.9C6, at 1:40 dilution and mouse anti-alpha Tubulin, Novus Biologicals, Littleton, CO, USA, cat. # NB100-690, at 1:5000 dilution). After washing, the membranes were probed for 1 h with a secondary antibody (a goat anti-mouse IgG (H + L) antibody, horseradish peroxidase conjugate, Invitrogen, cat. # G21040, at 1:10,000 dilution) at room temperature. The signals were detected using the ECL Western Blotting Analysis System (Amersham Pharmacia Biotech, cat. # RPN2108) on an Amersham Imager 600 instrument (GE Healthcare Bio-Sciences AB, Uppsala, Sweden).

## 3. Results

### 3.1. Oogenesis Disturbances in Mutants with an Altered 5′ Regulatory Region of the Notch Gene

**Figure 1:**
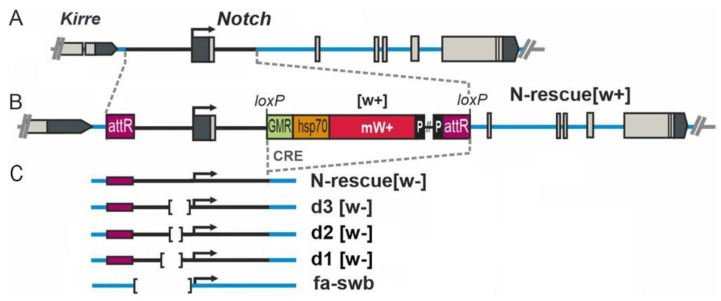
Locations of mutations in the 5′ region of the *Notch* gene. (**A**) The genetic map of the *Notch* locus. Grey boxes are exons, the thick black line is a 4 kbp fragment deleted in the founder fly strain, and the thick blue lines denote introns of the *N* gene. (**B**) As an example, the organization of the *Notch* gene in the N-resc[w+] fly line is shown, in which a full-size 4 kbp fragment from the 5′ region in the pGE-attB-GMR vector was utilized to reverse the deletion in the founder line through PhiC31-mediated integration. (**C**) The *mini-white* reporter gene and vector sequences ([w+]) were removed with the help of Cre recombinase to create the fly stocks where the engineered target region is flanked by attR and *loxP* sites (N-rescue[w−] and directed deletions of the 5′ region of the *Notch* gene). The position of the *N^fa-swb^* deletion is specified too.

We analyzed ovary morphology in all lines containing deletions in the *Notch* promoter region obtained in our previous work [15]. Positions of these deletions relative to each other and to the gene body are shown in Figure 1. Oogenesis disturbances were observed in the ovaries of females from lines resc[w+], d3[w+], and d3[w−] (Figure 1).

**Figure 2:**
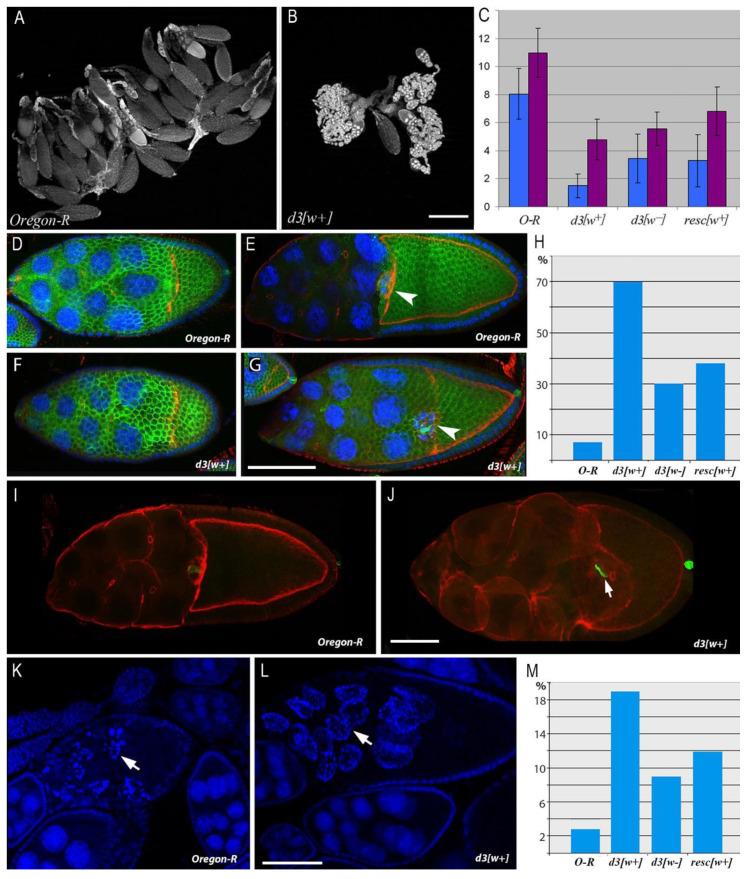
Comparative analysis of egg formation aberrations and oogenesis abnormalities caused by mutations in the 5′ region of *Notch*. (**A**,**B**). Ovary morphology in 3-day-old females of genotypes *Oregon-R* (**A**) and *d3[w+]* (**B**). (**C**). The egg number in the ovaries of 3-day-old (blue) and 5-day-old (pink) females. The bars denote the number of eggs in an ovary (mean ± SD; *n* = 40). Differences from the control (*Oregon-R*) are significant at *p* < 0.05. (**D**–**G**). Alteration of egg chamber (EC) morphology at stages 9–10A in the mutant background compared to the wild type. (**D**,**E**). In Oregon-R females, the ECs have the morphology characteristic of the stage 9 beginning (**D**) and of stage 10A (**E**). Border cells are situated at the boundary between the oocyte and nurse cells (arrow). (**F**,**G**). At the beginning of stage 9, EC morphology does not clearly differ between *d3[w+]* females and the wild type (**F**). At stage 10A (**G**), the mutant has abnormally small oocytes. Border cells reach the oocyte, just as in the wild type (arrow). Follicular cells remain in the area of the nurse cells and do not form the epithelium around the oocyte. Scale bar = 10 µm. (**H**). The histogram of percentages of abnormal ECs at stage 10A in the ovaries of mutant females. *n* = 80 for each cases, *p*-value: *d3[w+]* − *2.61 × 10^−44^*; *d3[w−]* − *2.52 × 10^−9^*; *resc[w+]* − *1.59 × 10^−14^*. (**I**,**J**). EC deformation at the 10B stage of oogenesis in *d3[w+]* females (**J**) in comparison with the wild type (**I**). Scale bar = 8 µm. (**K**–**M**). EC death in the oogenesis of *Oregon-R* and *d3[w+]* females. (**K**). Programed cell death at oogenesis stages 7–9 in *Oregon-R* females. The EC has signs of apoptosis (arrow: fragmented nuclei). (**L**). Ectopic death of the EC at stage 10B in the *d3[w+]* mutant. Scale bar = 10 µm. (**M**). The histogram of percentages of ECs dying at the 10B stage in the ovaries of the mutant females. *n* = 75 for each case, *p*-value: *d3[w+]* − *1.30 × 10^−8^*; d3[w−] − *0.004181*; *resc[w+]* − *0.000192*. F-actin (phalloidin), red; chromatin (DAPI), blue; the cell membrane of follicular cells (including polar) (Fasciclin3), green.

The resc[w+] line carries an insertion of an auxiliary element into the first intron of *Notch*. The d3[w+] strain harbors the same insertion in combination with a 255 bp deletion, which removes from this locus the binding sites of insulator proteins CTCF and CHROMATOR [15]. In the d3[w−] line, the insertion of the auxiliary element between *lox* sites is eliminated (Figure 1). We found that these mutations affect egg production. In the gonads of 3-day-old females of these lines, ECs at the stages of early and middle oogenesis proved to be predominant, and very few eggs were seen, whereas in the normal flies (Oregon-R line), mature eggs are prevalent in the ovaries, and there are only a few ovarioles with ECs (Figure 2A,B). Quantitative analysis indicated that in the ovaries, significantly fewer eggs are produced in 3–5-day-old mutant females than in the wild-type line (Figure 2C).

A detailed cytological analysis of oogenesis revealed that the mutant phenotype is the most pronounced in the ovaries of the d3[w+] line. By phalloidin staining of cortical actin filaments, the cell boundaries of the growing ECs were visualized at different stages of oogenesis. Normally, at the 10A stage of oogenesis, the EC has a number of specific morphological features. One half of its volume should be occupied by an oocyte covered with cylindrical epithelial cells, and the other half should be occupied by nurse cells covered with squamous epithelium cells. Additionally, at stage 10A, the migration of border cells ends, which stop when they reach the oocyte border and end up in the middle part of the EC (Figure 2E).

In d3[w+] females, many ECs at the 10A oogenesis stage contain abnormal oocytes: in contrast to wild-type *Drosophila*, their size is much smaller than the area of nurse cells. As in the wild type, the border cells are located at the boundary between the nurse cells and the oocyte, but due to the delay in the growth of the latter, they are displaced closer to the anterior end of the EC (Figure 2G). In contrast to the wild type, in mutants at this stage, a single-layer cylindrical epithelium around the oocyte and the squamous epithelium around the nurse cells do not form (Figure 2G). The morphology and position of follicular cells correspond to earlier stages of oogenesis, mainly the beginning of the ninth stage (Figure 2F,G). This phenotype is most strongly manifested in the d3[w+] mutant and to a lesser extent in resc[w+] and d3[w−]. Binomial tests showed that these differences are statistically significant (Figure 2H).

The percentage of abnormal ECs at the 10A stage is significantly higher in d3[w+] mutants than in the wild type (Figure 2H). Nonetheless, later, this defect is partially compensated (because the majority of ECs that have reached the end of the 10th stage possess normal morphology) and is detectable only in very few ECs having small oocytes. Such ECs look deformed and contain hypertrophied nurse cells, the volume of which has increased, probably due to impaired transport of cytoplasmic contents from these cells to the oocyte (Figure 2J). At this stage of oogenesis, the mutants exhibit a high percentage of dying ECs. In case of normal ovary development, the death of ECs at the 10th stage of oogenesis is a rare event because defective ECs are eliminated earlier, at stages 7–9. In the ovaries of Oregon-R female flies at the 10th stage of oogenesis, there are only 3% of ECs with signs of cell death, as evidenced by chromatin fragmentation after staining with DAPI (Figure 2K,L). In mutants resc[w+] and d3[w+], this index is several times higher (Figure 2M). The alterations of structure and morphology in some ECs in the mutants are apparently harmful because they induce ectopic cell death in late oogenesis.

We performed a number of immunostaining experiments on resc[w+] and d3[w+] ECs with specific antibodies. For instance, mitosis in follicular cells was analyzed with antibodies to phosphorylated H3 histone. The polarity of the oocyte was investigated by staining with antibodies to proteins Gurken and orb as well as antibodies to microtubules and microtubule organizing centers α-Tubulin and Asterless. The location of the oocyte nucleus depending on the stage of oogenesis was examined with the help of an antibody against the Lamin DmO protein, which is specific to the nuclear envelope. The results of these experiments indicated that mutations resc[w+] and d3[w+] do not influence oocyte polarization and mitosis in follicular cells (Figure S1).

### 3.2. The Notch Expression Level in the Ovaries of the Mutant Females

**Figure 3:**
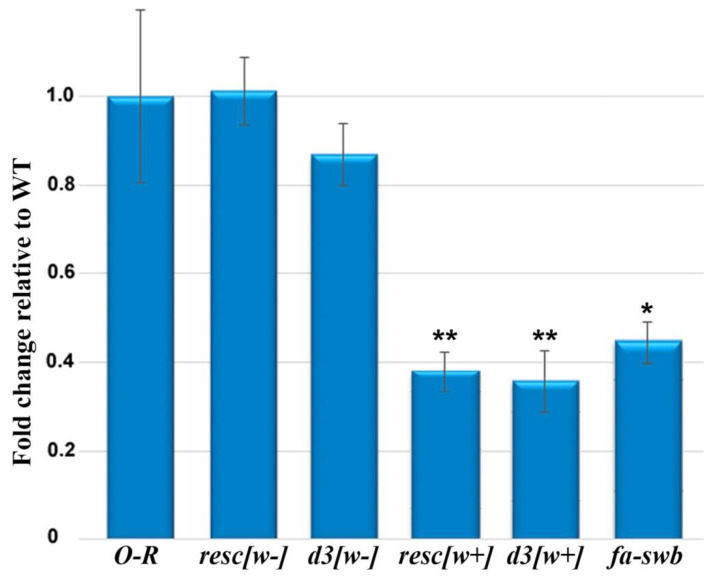
Transcription of the *Notch* gene as measured by RT-qPCR in wild-type and mutant ovaries of imago females. The *Zfrp8* gene was used for normalization as an endogenous control. Error bars represent standard error of the mean from three replicates (*n* = 3). To evaluate the significance of differences between samples, Student’s *t* test was performed (*n* = 3). * *p* < 0.05, ** *p* < 0.01.

We quantified *Notch* gene transcription in the ovaries from flies of lines Oregon-R, resc[w+], resc[w−], d3[w+], d3[w−], and *N^fa-swb^*. The results are displayed in Figure 3.

There are no differences in the *Notch* gene transcription level between the ovaries of resc[w−] females in which the auxiliary element between *loxP* sites in the first intron was completely removed [15] and Oregon-R ovaries, consistent with the data on the normal morphology of ovaries in these females. In the ovaries of resc[w+] and d3[w+] females, the magnitude of this gene’s expression turned out to be approximately three times lower than that in the control. In females of the d3[w−] line, which features the d3 deletion without the auxiliary element in the first intron [15], only slightly weaker *Notch* transcription was observed. For comparison, we determined the level of this transcription in the *N^fa-swb^* line, which represents the classic *Notch* allele consisting of a deletion that is located immediately upstream of the *Notch* core-promoter region and spans a DNA segment of 880 bp [19]. Expression of the *Notch* gene in the ovaries of females of this line is also low and comparable to the transcription level of this gene in the ovaries from lines d3[w+] and resc[w+] (Figure 3).

**Figure 4:**
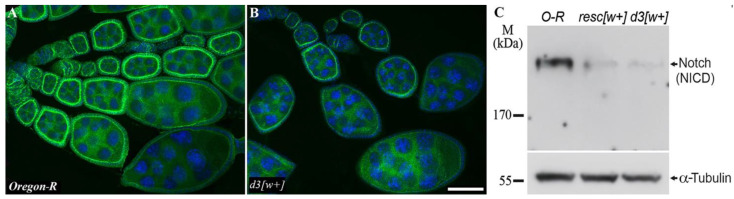
Protein localization and expression in the oogenesis of the mutants and Oregon-R flies. (**A**,**B**). Staining with the anti-Notch intracellular domain antibody uncovered no difference in the Notch localization between Oregon-R (**A**) and d3[w+] ovaries (**B**), but in d3[w+] ovaries, the intensity of staining is weaker and close to the background. The scale bar is 10 µm. (**C**). Western blot analysis of Notch protein expression in ovaries of Oregon-R (control), resc[w+], and d3[w+] imago females. Staining with antibodies to tubulin serves as a loading control.

For a more detailed analysis of this gene’s activity in the ovaries, the localization pattern of the Notch protein during oogenesis was determined by immunostaining with antibodies to the extracellular and intracellular domains of this protein. In females of the Oregon-R line, the antibody staining resembles that described in other works [8,20]: the protein is clearly visualized in the area of the apical membrane of follicular cells from early stages of oogenesis (germarium) up to stage 7. After that, at the seventh stage, the color intensity declines noticeably (Figure 4A).

The staining patterns are similar between the antibodies to the extracellular and intracellular domains. In d3[w+] females, the pattern of protein localization during oogenesis does not differ from that of the wild type, but the staining looks less intense and is only slightly different from the background (Figure 4B). Notable underexpression of the Notch protein in lines d3[w+] and resc[w+] was confirmed by Western blot analysis (Figure 4C, Figure S2).

## 4. Discussion

According to our data, in the ovaries of resc[w+] females and d3[w+] females, the *Notch* gene transcription level is significantly lower than that in the wild type. The resc[w+] line carries an insertion of auxiliary elements of the transgenic construct in the first intron of the *Notch* gene, and this insertion manifests itself as a hypomorphic mutation. In the homozygous state, it gives rise to a weak *Notch*-like phenotype: small nicks on the wings in males and partially rough eyes. In a trans-heterozygote with the founder deletion removing the 5′ regulatory region of the gene and a part of the first intron, this mutation causes multiple phenotypic defects in *Notch* target tissues [15]. The lowered *Notch* gene transcription in the ovaries is exclusively due to the insertion of auxiliary elements because in the resc[w−] line—in which the insertion is removed at *loxP* sites and the sequence of the 5′ region of the gene is restored to normal—the gene transcription level is not altered (Figure 3). Mutations in the introns of the gene that affect fly development have also been obtained and characterized before; these include, for example, *facet* group mutations caused by insertions of *copia*-like mobile elements into the second intron of the gene [21]. All mutations in this group disarrange the correct formation of the facet eye. The finding that introns contribute to the regulation of *Notch* activity was confirmed in transgenic experiments with constructs containing either the full-length genomic copy of the gene or minigene versions that lack most introns [19].

Our data on the low transcription of *Notch* in the ovaries of the mutant females suggest that there are some tissue-specific regulatory elements in the introns of the *Notch* gene that interact with functional sequences of the promoter region. In particular, such elements can be enhancers, which, according to evidence obtained in recent years, are found in all long introns of this gene, and many of these enhancers are active in ovaries [22].

In addition to the insertion into the first intron, the d3[w+] line is characterized by a deletion that removes a 255 bp fragment, including the insulator sequence in the promoter region of the gene [15]. This mutation leads to significantly lower expression of the *Notch* gene in the ovaries of the mutant females and disrupts EC development in middle oogenesis. The mutation does not cause female sterility but rather slows down oogenesis and diminishes the number of eggs generated.

Requirements for Notch signaling during oogenesis have been analyzed previously using conditional alleles of *Notch* and *Notch*-mutant clones. Temperature-sensitive alleles of *Notch* and null allele homozygous clones of follicular cells give an EC fusion phenotype in which a single follicular epithelium surrounds several cysts [8,10]. Moreover, *Notch* hypofunction results in hyperplasia and a loss of epithelial characteristics among posterior follicle cells [23,24]. Aberrant follicle cell differentiation and behavior in the *Notch* misregulation background are also observed in vps26 mutant clones. In this case, Notch ligand Delta traffic was disrupted leading to impaired intercellular signaling between the germline and the follicle cells [25].

In the present work, we demonstrated that the main abnormalities in the ovaries of resc[w+] and d3[w+] females alter follicular cells. In the mutants that we described, a single-layer cylindrical epithelium around the oocyte and the squamous epithelium around the nurse cells do not form at stage 10. Instead, the morphology and arrangement of follicular cells correspond to earlier stages of oogenesis, mainly the beginning of the ninth stage. Notch signaling is repeatedly required during follicular-cell development. *Notch* is important for the genesis of the two follicular cell lineages: the stalk/polar cell lineage and the epithelial follicular cell lineage [7,8,26]. Subsequently, during stage 6, *Notch* is necessary throughout the follicular cell epithelium for the switch from mitotic cell divisions to endoreplication [10,11]. Loss of Notch in the epithelial cells themselves blocks the differentiation of these cells and causes their arrest in an immature state. Furthermore, the mutant cells do not switch from the mitotic cell cycle to the endocycle at stage 6 and continue to divide for the rest of oogenesis [10]. According to our findings, the *Notch* underexpression induced by the insertion into the first intron does not affect the formation of polar and stalk cells and apparently does not disrupt the switch from mitotic cycles to the endocycle in follicular cells. On the other hand, this deficiency has an impact on the terminal stage of follicular-cell differentiation, i.e., the correct migration of follicular cells and the assembly of the columnar epithelium around the oocyte. These data are consistent with the results of the work of M. Grammont, who showed that *Notch* is necessary for stretched cells flattening and the main body follicular cells displacement at stage 9 of oogenesis. The author showed that these processes depend on correct adherens junction remodeling, which is regulated by some *Notch* target genes [27].

In resc[w+] and d3[w+] females, abnormal oocytes, whose volume was significantly less than the area of nurse cells, formed in many ECs at the 10A stage of oogenesis. In later stages, such ECs contained hypertrophied nurse cells. It is possible that the *Notch* underexpression gives rise to disturbances in the processes associated with slow selective transport from nurse cells to the oocyte. This transport determines the growth and increase in the volume of the latter. On the other hand, slow oocyte growth may cause abnormal migration of follicular cells.

Thus, we showed that the insertion of an auxiliary element into the first intron of the *Notch* gene alone or in combination with a deletion of the insulator sequence significantly weakens this gene’s expression in the ovaries and results in various disorders of oogenesis, such as an oocyte growth delay, impaired migration of follicular cells, and anomalous epithelium formation. At the same time, the combination of this insertion with deletion d1 or d2 (Figure 1) also reduces the level of *Notch* expression but does not disrupt the normal course of oogenesis (data not shown). This observation points to the complex nature of interactions of various sequences of the *Notch* gene promoter region with possible functional sites located in introns of this gene. A recent study on the structural and functional organization of this gene containing targeted (CRISPR/Cas9-induced) mutations obtained in *Drosophila* cell cultures [28] confirms these suppositions.

## 5. Conclusions

We demonstrate new effects of the *Notch* gene on oogenesis by means of our previously generated mutations. Mutant lines carry an insertion of auxiliary elements of a transgenic construct in the first intron of *Notch* and a series of directed deletions affecting possible functional sites of the 5′ regulatory region. We show that some of these mutations influence *Drosophila* oogenesis. The insertion of the auxiliary elements of the transgenic construct into the first intron of *Notch* either alone or in combination with a deletion of an insulator sequence diminishes the expression of this gene in the ovaries. As a consequence, the formation of ECs is disturbed in middle oogenesis. These abnormalities alter cellular processes that ensure the growth of oocytes and assembly of the specialized epithelium around them.

## Supplementary Materials

The following supporting information can be downloaded at: https://www.mdpi.com/article/10.3390/jdb10030032/s1, Figure S1: Immunostaining experiments on d3[w+] and Oregon-R ECs with specific antibodies; Figure S2. Western blot analysis of Notch protein expression in ovaries of Oregon-R (control), resc[w+], and d3[w+] imago females.
