# Supplementary material for: New Mutations in the 5′ Region of the Notch Gene Affect Drosophila melanogaster Oogenesis
Source: J Dev Biol. 2022 Aug 9;10(3):32. doi: 10.3390/jdb10030032 (PMC9397085; doi:10.3390/jdb10030032)
Supplement: Supplementary file 1 [file jdb-10-00032-s001.zip › jdb-1785234-supplementary.pdf]

Supplementary material

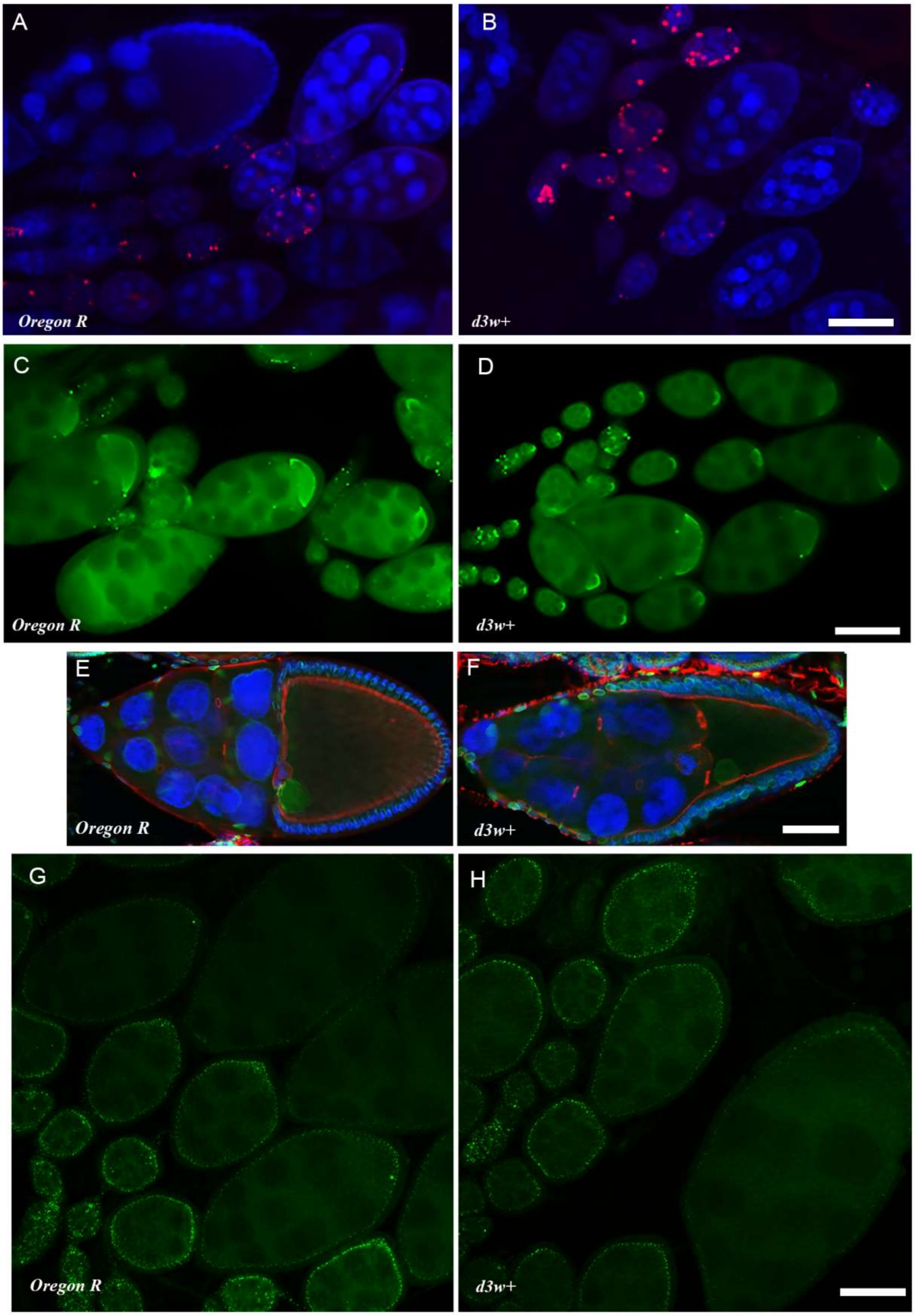

Figure S1. Immunostaining experiments on d3[w+] and Oregon-R ECs with specific antibodies.

A, B. Detection of mitotically dividing cells in the oogenesis of the *OregonR* (**A**) and *Notch* mutant (**B**) ovaries using antibody to phospho- (p-)H3 histone marked mitotic chromosomes. No differences were found between wild type and *Notch* mutation. In both cases, mitoses stop before the 6th stage of oogenesis, and thus the mutations do not affect the timely mitoses completion. DNA (DAPI) - blue, p-H3 histone - red. Scale bars: 10 μm

C, D. Localization of the oocyte-specific Orb protein identified using anti-Orb antibodies in *Oregon R* (**C**) and *Notch* mutant (**D**) ovaries. Orb is RNA-binding protein required establishment of oocyte polarity, interacts with and regulates Oskar mRNA translation. The anti-Orb immunostaining pattern in the mutant ovary does not differ from the wild type. Orb - green. Scale bars: 10 μm.

E, F. Visualization of the oocyte nuclei in *Oregon R* (**E**) and *Notch* mutant (**F**) ovaries with the help of antibodies to the Lamin Dm0 protein. The localization of the nucleus in the mutant's egg chamber the same as in wild type's chamber. Lamin Dm0 – green. Scale bars: 3 μm.

G, H. Anti-Asterless labeled centrioles in *Oregon R* (**G**) and *Notch* mutant () ovaries. The localization of centrioles in the oocyte and follicular cells in the mutant does not differ from the localization of centrioles in control line *Oregon R*. Centrioles (Asterless) – green. Scale bars: 12 μm.

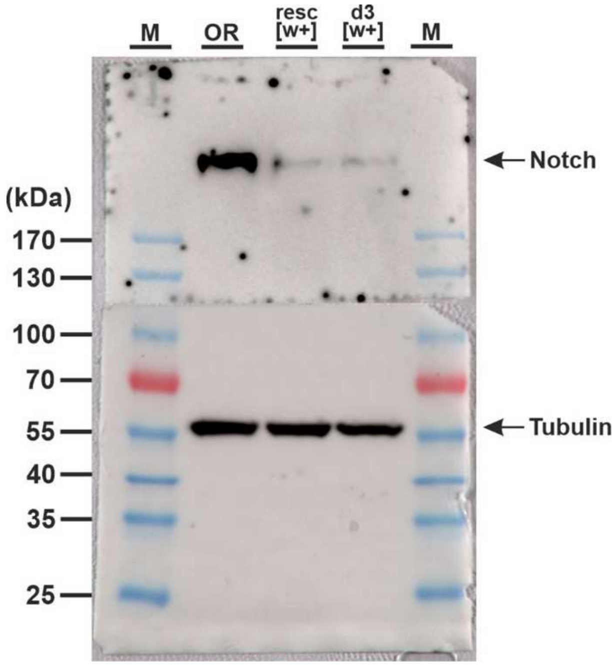

Figure S2. Western blot analysis of Notch protein expression in ovaries of Oregon-R (control), resc[w+], and d3[w+] imago females. Females were raised at 25°C. A decrease in the intensity of anti-Notch (Notch intracellular domain) immunoreactive bands (the arrow indicates the full-length ~300 kDa Notch product) was observed only for resc[w+] and d3[w+] female ovarian samples. Staining with antibodies to alfa-Tubulin serves as a loading control; M – prestained protein ladder.
